# A peristomal plaque of sudden occurrence

**DOI:** 10.1002/ccr3.3939

**Published:** 2021-03-02

**Authors:** Michelangelo La Placa, Diego Abbenante, Massimiliano Pazzaglia, Ambra Di Altobrando

**Affiliations:** ^1^ Dermatology Division IRCCS Sant'Orsola Policlinico University of Bologna Bologna Italy

**Keywords:** colostomy bag, Koebner phenomenon, peristomal skin, psoriasis

## Abstract

Koebner phenomenon regards the formation of a psoriatic lesion after a trauma, including tattoo, insect bite or other injuries. Although this manifestation is not specific for psoriasis, physicians should be aware because early recognition may be helpful in making the diagnosis when present.

## Graphical abstract

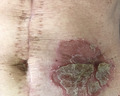

## 1 INTRODUCTION

**FIGURE 1:**
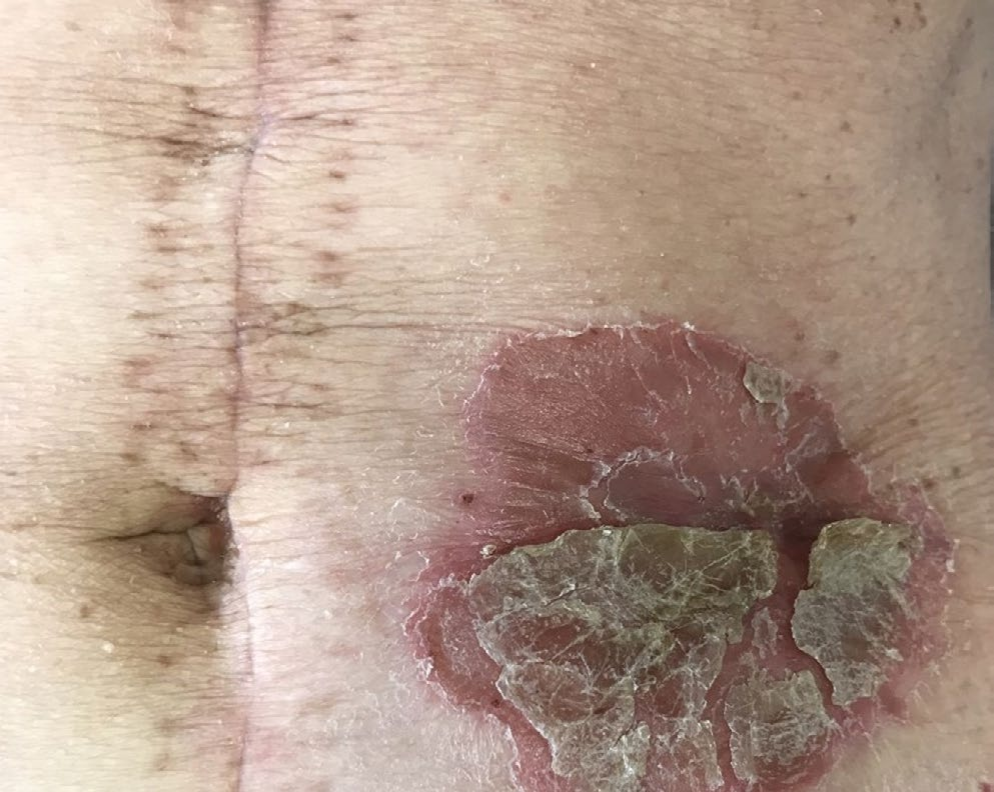
Well‐defined psoriatic plaque around the stoma

A 69‐year‐old woman presented with a 10‐day history of an asymptomatic peristomal plaque. A large erythematous scaly plaque, 10 cm. in diameter, with sharply demarcated borders, was observed on the left abdominal side, around the stoma (Figure 1).

What is you diagnosis?

## 2 DISCUSSION AND OUTCOMES

The patient had colostomy 2 years before to treat a colonic perforation due to a complication following aortic abdominal aneurysm. She reported mild psoriasis for approximately 10 years without specific treatment, but using topical steroids occasionally. The diagnosis of psoriasis following Koebner phenomenon (KP) was made.^1^, ^2^

Koebner phenomenon, also known as isomorphic response, describes the sudden occurrence of an inflammatory dermatosis, including psoriasis, lichen planus, vitiligo, or lupus erythematosus, on previously unaffected skin after traumatic stimuli (Table 1). This phenomenon has been reported after tattoo, burns, frostbite, insect bites, surgery, wounds or in old scars of sarcoidosis and smallpox vaccination (Table 2). The pathogenetic mechanisms of KP are still unclear, as to whether these manifestations are limited to the skin and mucosae, or may arise on internal organs is further questioning.^3^ All physicians should be aware that traumatic episodes must be avoided in people with underlying immunological diseases.

**TABLE 1:** Cutaneous manifestations of Koebner phenomenon

Psoriasis
Lichen planus
Vitiligo
Lupus erythematosus (discoid)
Erythema multiforme
Molluscum contagiosum
Warts

**TABLE 2:** Causes of Koebner phenomenon

Burns
Insect bites
Tattoo
Surgical procedures
Friction/excoriation
Laceration/scarification

## CONFLICT OF INTEREST

The authors have no conflict of interest.

## AUTHOR CONTRIBUTIONS

MLP, DA, MP, and ADA: All authors have participated in the work, giving substantial contributions to conception and design, acquisition of data, drafting the manuscript, and final approval of the version to be published.
